# Vaccination as a Strategy for Cardiovascular Prevention: Closing a Gap in Clinical Practice

**DOI:** 10.31083/RCM54010

**Published:** 2026-09-11

**Authors:** Elvira García-de-Santiago, Alberto García-Lledó

**Affiliations:** ^1^Department of Biomedical Sciences, Universidad de Alcalá, 28805 Alcalá de Henares, Madrid, Spain; ^2^Department of Medicine, Universidad de Alcalá, 28805 Alcalá de Henares, Madrid, Spain; ^3^Department of Cardiology, University Hospital Príncipe de Asturias, 28805 Alcalá de Henares, Madrid, Spain

**Keywords:** myocardial infarction, acute coronary syndrome, myocardial ischemia, infections, vaccines, influenza, human, Streptococcus pneumoniae, COVID-19, respiratory syncytial viruses, herpes zoster

## Abstract

Prevention of the consequences of atherosclerotic disease has traditionally focused on controlling individual risk factors such as dyslipidaemia, hypertension and diabetes. In recent years, the term residual risk has been used to describe the persistence of cardiovascular events despite the optimal management of these classical risk factors, with particular attention given to inflammation. Far less attention, however, has been paid to environmental risk factors, which affect entire populations and may account for a substantial proportion of temporal variations in event incidence, as well as the marked seasonal fluctuations in hospital admissions for cardiovascular disease that periodically place considerable pressure on healthcare systems. Temperature, air pollution and infectious diseases—particularly respiratory infections—may help explain why an individual with a given baseline risk experiences an event in one month rather than another. Some environmental factors may promote the development of classical risk factors, whereas others may contribute directly to atherosclerosis. All of them may act either as triggers for acute ischemic events or as destabilising factors in previously stable patients, for example those with heart failure. Infections, especially those of respiratory tract, play a key role in the natural history of cardiovascular disease. Growing evidence indicates that preventing infections through vaccination can reduce the risk of acute ischemic events, hospitalisation and death. Nevertheless, vaccination coverage for many infections, including influenza and Coronavirus disease 2019 (COVID-19), remains low. In addition, pseudo-scientific campaigns continue to spread misinformation about vaccines, thereby reducing patient adherence. Leading cardiology societies now recognise vaccination against certain infections as an evidence-based strategy for cardiovascular prevention. It is therefore essential that this information be widely disseminated among healthcare professionals, patients and the general public. Of all the available measures to control environmental risk factors, vaccination is the only intervention that clinicians can directly implement in routine clinical practice. Vaccination recommendations should therefore be regarded with the same importance as advice on diet, exercise and smoking cessation.

## 1. Introduction: the Concept of Environmental Risk

Cardiovascular diseases are the leading cause of death in developed countries. Among these, ischemic heart disease (IHD) accounts for a significant proportion of the burden [1]. IHD is closely linked to coronary atherosclerosis and therefore to the risk factors for atherosclerotic disease (cardiovascular risk factors, CRFs). The clinical management of cardiovascular risk typically involves analysing each individual’s CRFs using scoring systems that differ according to the level of risk in the population.

This individual-based approach does not explain why a patient with a given risk experiences an acute event at a specific time rather than another. Both population mortality and the epidemiology of numerous diseases, including IHD, exhibit seasonal cycles and other circadian rhythms in the occurrence of acute cases [2]. The epidemiological concept of ‘winter mortality’ [3,4] (Fig. 1, Ref. [4]) has its counterpart in the epidemiology of acute coronary syndrome (ACS), whose incidence rises sharply during cold periods [5] in countries with cold and temperate climates, a finding that cannot be explained by classic CRFs.

**Fig. 1.**
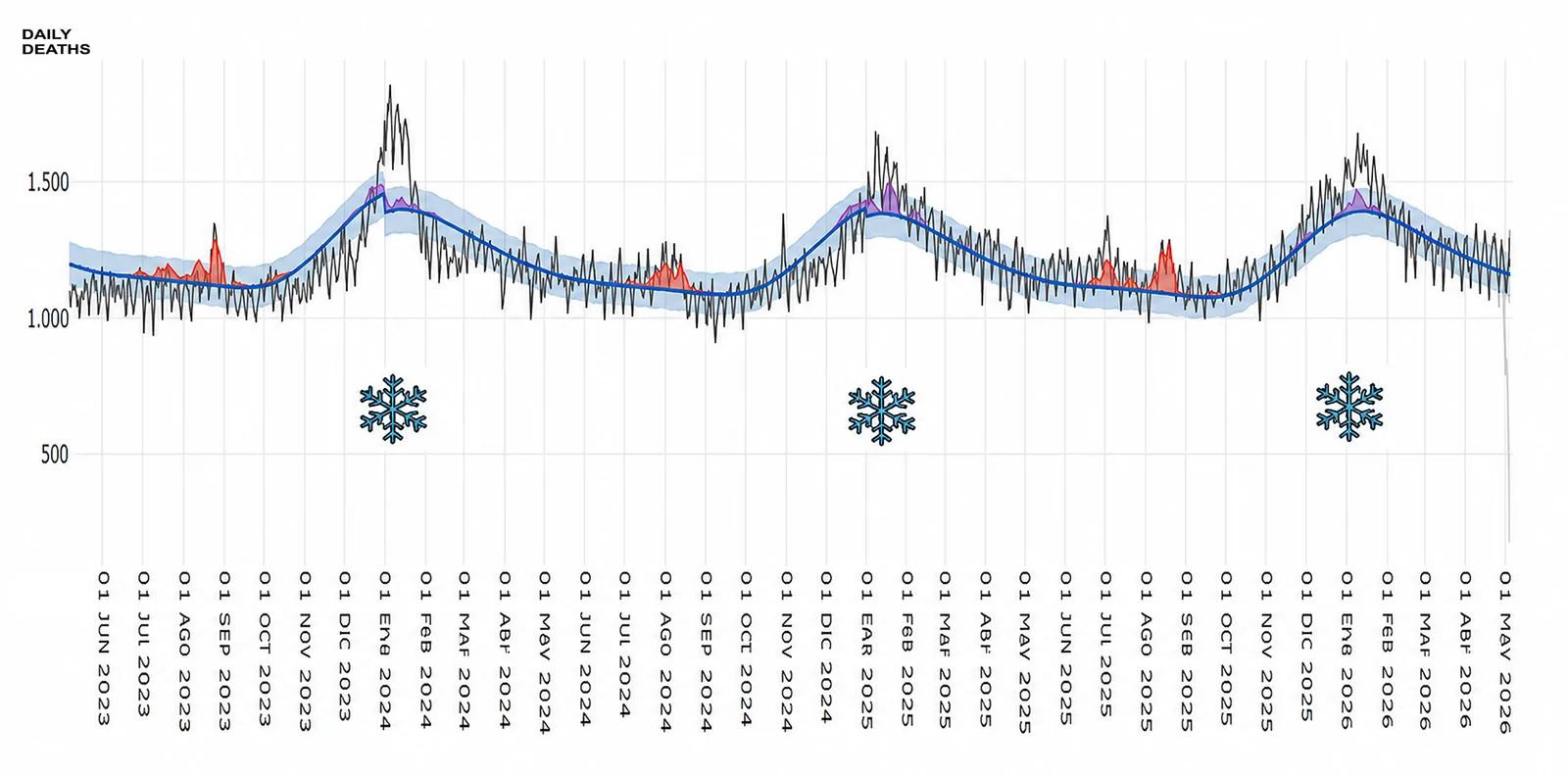
**Curve of mortality in Spain (June 2023 to May 2026)**. A cyclical pattern in mortality is observed, peaking during the winter months (Data from the Daily Mortality Monitoring Panel-MoMo.  https://momo.isciii.es/panel_momo/. Institute of Health Carlos III, Madrid, Spain) [4].

Focusing on acute coronary syndrome (ACS), the cold and seasonal respiratory infections could be considered responsible for this finding. In countries with a temperate climate, most respiratory infections follow a seasonal pattern that coincides with the cold periods of autumn and winter. It has been shown that both factors increase the risk independently of each other [5]. The interactions between the environment and acute cardiovascular events may be even more complex. An increased risk of ACS has been reported in association with environmental pollution [6] and the number of daylight hours per day. These two factors, in turn, show clear seasonal variation: daylight hours are shorter in winter, and levels of gaseous pollutants are higher during cold periods. Furthermore, low income and low levels of education are also associated with an increased risk of cardiovascular disease [7], and vaccination rates are lower in populations with low educational attainment [8]. Any analysis of the relationship between all these environmental variables and the risk of cardiovascular events must consider the complexity of these interactions. Environmental risk factors can trigger acute events in patients with pre-existing risk factors, which could explain the higher incidence of acute events during specific periods, despite individual risk remaining relatively stable over time [2,5]. In the case of cold, for example, the adrenergic response could explain both an increased probability of rupture of pre-existing atheromatous plaques and elevated platelet aggregability in response to such ruptures [9]. Most importantly, infections, particularly but not limited to respiratory tract infections, can trigger acute ischaemic events through mechanisms that will be detailed later, which include atherosclerotic plaque vulnerability, immunothrombosis and metabolic disbalance [5].

From a clinician’s perspective (Fig. 2), there is little we can do in our daily practice to prevent the effects of cold or pollution. However, an understanding of environmental risk factors should make us aware of the need to make use of all available tools to reduce the risk of IHD associated with infection, namely vaccines. The aim of this narrative (non-systematic) review is to highlight the importance of using them.

**Fig. 2.**
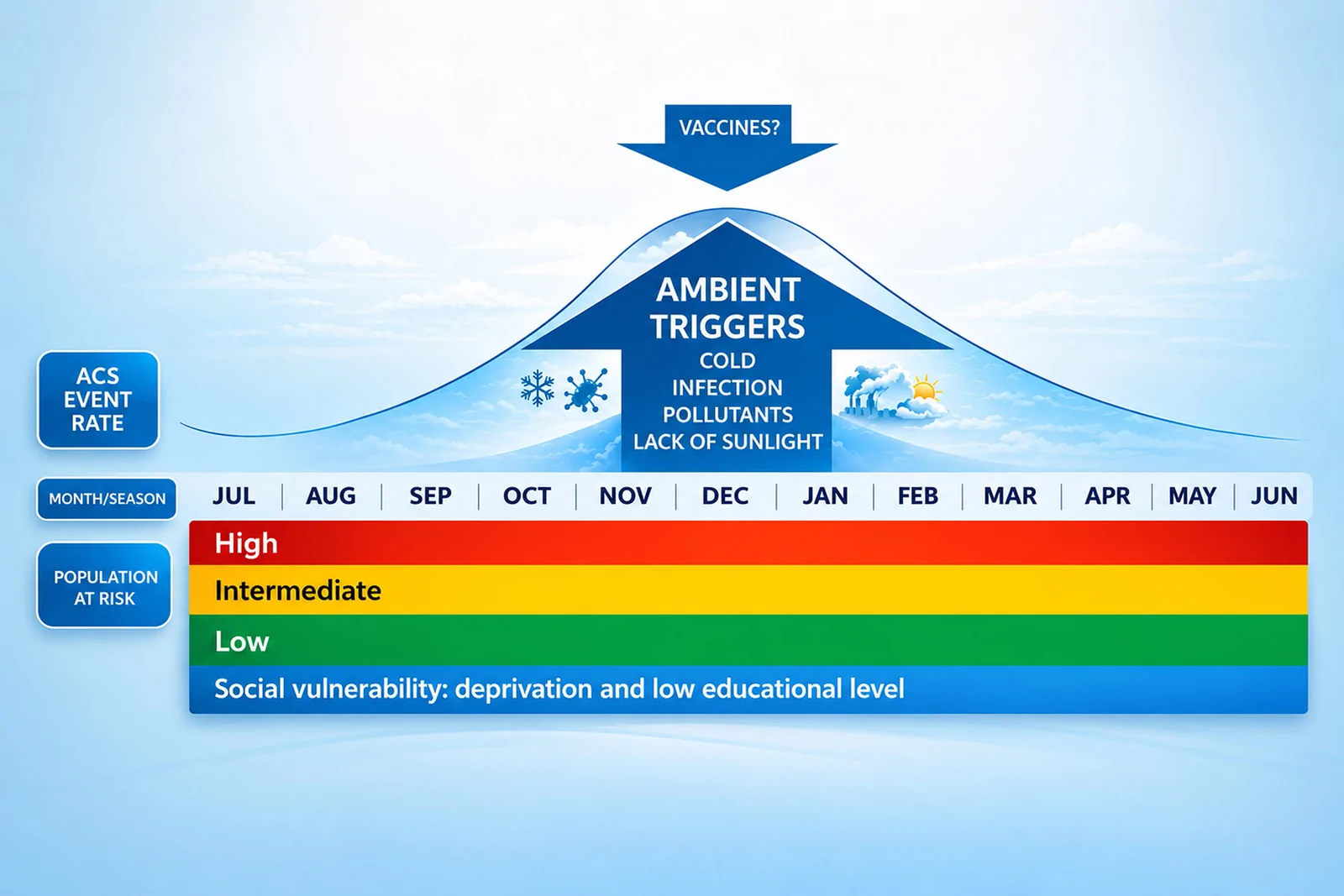
**Schematic representation of the interaction between ambient risk factors and the risk of developing an ischaemic event, and the potential benefit of vaccines**. ACS, acute coronary syndrome.

## 2. Infection and the Risk of Ischaemic Heart Disease

The association between infection and an increased risk of developing an ACS has been recognised for almost 100 years. A case-series study by Smeeth et al. [10] conducted using the United Kingdom General Practice Research Database found an increased risk of acute myocardial infarction (AMI) following a respiratory infection, with an incidence ratio of 4.95 (95% confidence interval [CI], 4.43 to 5.53). This risk peaked three days after the infection, decreased progressively in the following weeks, and was similar for first-time and recurrent events. The same was observed regarding the relationship between respiratory infection and stroke episodes. Also, urinary infections were associated with a lower but significant risk. Notably, the authors found no increase in ischemic events following vaccination against influenza, tetanus, or pneumococcus, which provided initial evidence that these vaccinations were safe even causing a transient inflammatory response. Analysis of the Danish national registry between 1987 and 2018 also shows a significant increase in the risk of AMI during the 30 days following admission for an infectious condition caused by various pathogens in different locations [11]. The greatest increase was observed for pneumonia (relative risk [RR] of 3.39, 95% CI 3.15–3.75 compared with controls), followed by urinary tract infections (RR of 2.44, 95% CI 2.21–2.70) and bone and soft tissue infections (RR of 1.84, 95% CI 1.45–2.33). Similarly, Staphylococcus aureus bacteraemia has been reported to be associated with an AMI risk up to 35 times higher than that of the control group in the days following the event [12].

Respiratory infections, whether viral or bacterial, are recognised as being associated with a higher risk of ACS than other infections [10,11]. Most of the evidence relates to those caused by influenza, pneumococcus, COVID-19 and, more recently, respiratory syncytial virus (RSV) [13,14,15,16,17,18,19] although it has also been observed with other viruses, such as adenovirus, human metapneumovirus and rhinovirus. Furthermore, it appears that the risk of ACS increases with the severity of the infection and is higher in cases of sepsis-associated pneumonia than in upper respiratory tract infections [11].

It also seems that the length of time during which the risk rises depends on how severe the infection is. In general, an increased risk of AMI has been reported during the week following a rise in the incidence of serologically confirmed viral respiratory infections [16,17], as well as for influenza-like illnesses (ILI) [5]. However, for pneumonia, the increased risk can persist for up to one year after infection [14]. This appears to be independent of the fact that people with CRFs have a higher risk of developing pneumonia due to health conditions such as age and diabetes [15]. Something similar could occur following the spread of COVID-19 [18]. However, the higher incidence and lethality of this disease in people with CRFs [19] and the significant variation in COVID-19 infection presentation during and after the pandemic make it difficult to understand its current interaction with cardiovascular disease.

From a theoretical standpoint, there are five potential mechanisms through which infections may elevate the risk of IHD. These include increasing the likelihood of developing CRFs, directly contributing to atherosclerosis, triggering atheromatous plaque rupture, facilitating thrombosis on ruptured plaques, or causing an imbalance between oxygen supply and demand in the myocardium. The first two mechanisms would act chronically, leading to atherosclerosis, while the latter three would act as triggers for ACS (Fig. 3).

**Fig. 3.**
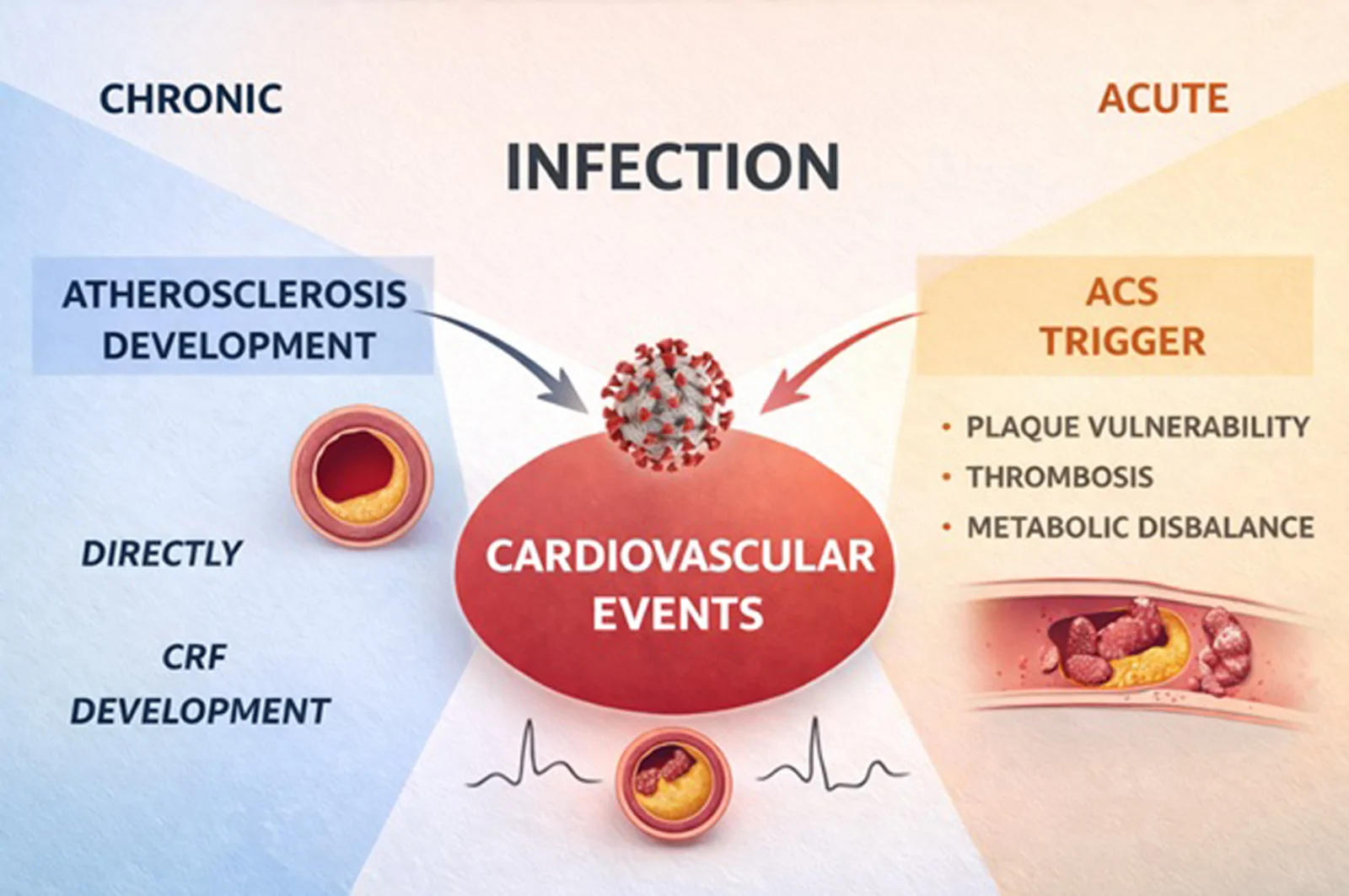
**Potential mechanisms by which infection may increase the risk of ACS**. In the long term, it could cause atherosclerosis either directly or indirectly by triggering or exacerbating a risk factor. In the short term, it could act as a trigger for plaque rupture, facilitating thrombosis or causing metabolic imbalance in the myocardium. ACS, acute coronary syndrome; CRF, cardiovascular risk factor.

### 2.1 Increased Risk of Atherosclerosis

Infections, particularly chronic ones, have been linked to the development of atherosclerosis. Certain human herpesviruses have been identified in atherosclerotic tissue obtained during post-mortem examinations [20], and it has been observed that these viruses can induce atherosclerosis in animal models. The presence of Chlamydia pneumoniae, Helicobacter pylori and Cytomegalovirus (CMV) has been detected in non-atherosclerotic arteries of patients with coronary artery disease [21]. In those subjects in whom the pathogens’ DNA was shown, inflammatory activity was found to be higher. Individuals infected with human immunodeficiency virus (HIV) have a 1.5-fold higher risk of AMI compared with seronegative individuals. The underlying mechanisms are not fully understood, but are likely to include a combination of the adverse cardiovascular effects of anti-HIV drugs [22] and the increased inflammatory activity associated with the presence of the virus, not to mention the fact that co-infection with CMV and herpes is highly prevalent in people with HIV [22].

Infections have also been linked to an increased likelihood of developing CRFs. Severe infections and chronic viral infections appear to increase the risk of developing type 2 diabetes [23]. CMV infection, which is highly prevalent in the adult population, has been proposed as a factor associated with the risk of hypertension. Herpesviruses, including CMV, encode viral G-protein-coupled receptors (vGPCRs) that could be associated with increased blood pressure, and have been proposed as therapeutic targets for the management of this risk factor [24]. Chronic Helicobacter pylori infection appears to be associated with a higher prevalence of dyslipidaemia, and its eradication may be associated with a reduction in lipid levels [25].

In summary, there is evidence supporting the hypothesis that various infections may be associated with an increased risk of developing atherosclerosis, both directly and through the induction of cardiovascular risk factors.

### 2.2 Infection as a Trigger for Myocardial Infarction

The fourth universal definition of myocardial infarction [26] recognises five types, two of which are relevant to the study of the relationship between infection and ACS: type I and type II infarctions. Both types involve myocardial necrosis of non-iatrogenic ischemic origin. In the first case, this is due to an arterial obstruction caused by the rupture of a coronary atheromatous plaque, on which an occlusive thrombus then forms. In the second case, it is due to an imbalance between the supply of oxygen to the myocardium and the demand for it. From a clinical perspective, infarcts are classified as either ST-segment elevation myocardial infarction (STEMI) or non-ST-segment elevation myocardial infarction (NSTEMI) due to the distinct management strategies required for each type [27]. STEMIs are usually type I infarcts and type II infarcts predominantly present as NSTEMIs, although this relationship is not fixed. Up to 23% of type II infarcts present with ST-segment elevation. Conversely, the plaque rupture that causes a type I infarct may present as a STEMI if the vessel is completely occluded and as a NSTEMI if the occlusion is incomplete [27]. Therefore, in clinical practice, cardiac catheterisation (or autopsy) can be necessary to distinguish between type I and type II myocardial infarction.

In the absence of a respiratory infection, STEMI accounts for 25% of all heart attacks [28]. However, in individuals with respiratory infections, this figure drops to 15% for non-influenza infections and to 10% for influenza cases [29]. Since respiratory infections increase the total number of heart attacks [13,14,16,17,18], the increased proportion of NSTEMIs suggests a greater role for the mechanisms characteristic of type II myocardial infarction (hypoxaemia and tachycardia) and a lesser role for plaque rupture as the primary mechanism. This increase could also be due, at least in part, to the higher incidence of NSTEMI in older people, who are more susceptible to severe infections [28]. While the mechanisms leading to an imbalance between oxygen supply and demand (type II AMI) are a significant factor in the increased risk of AMI due to infection, influenza has been shown to be associated with an increase in type I AMI, with plaque rupture demonstrated during cardiac catheterisation [5]. It is also possible that some episodes classified as NSTEMI due to transient troponin elevation during an infection may be caused by other factors. For example, up to 2.9% of patients with influenza have been reported to have elevated serum troponin levels, and half of these patients did not receive a final diagnosis of ACS [29,30]. An elevation in myocardial necrosis biomarkers can be caused by myocarditis, sepsis or impaired renal function, and may lead to an incorrect ACS diagnosis, particularly in patients with severe infection or overt sepsis. This must be taken into particular consideration when analysing the incidence of myocardial infarction during the COVID-19 pandemic, when a large number of critically ill patients with shock from various causes were admitted in a very short period of time. Fig. 4 illustrates the pathways through which acute infection can trigger a type I or type II myocardial infarction, although in everyday clinical practice both are most likely to occur in combination.

**Fig. 4.**
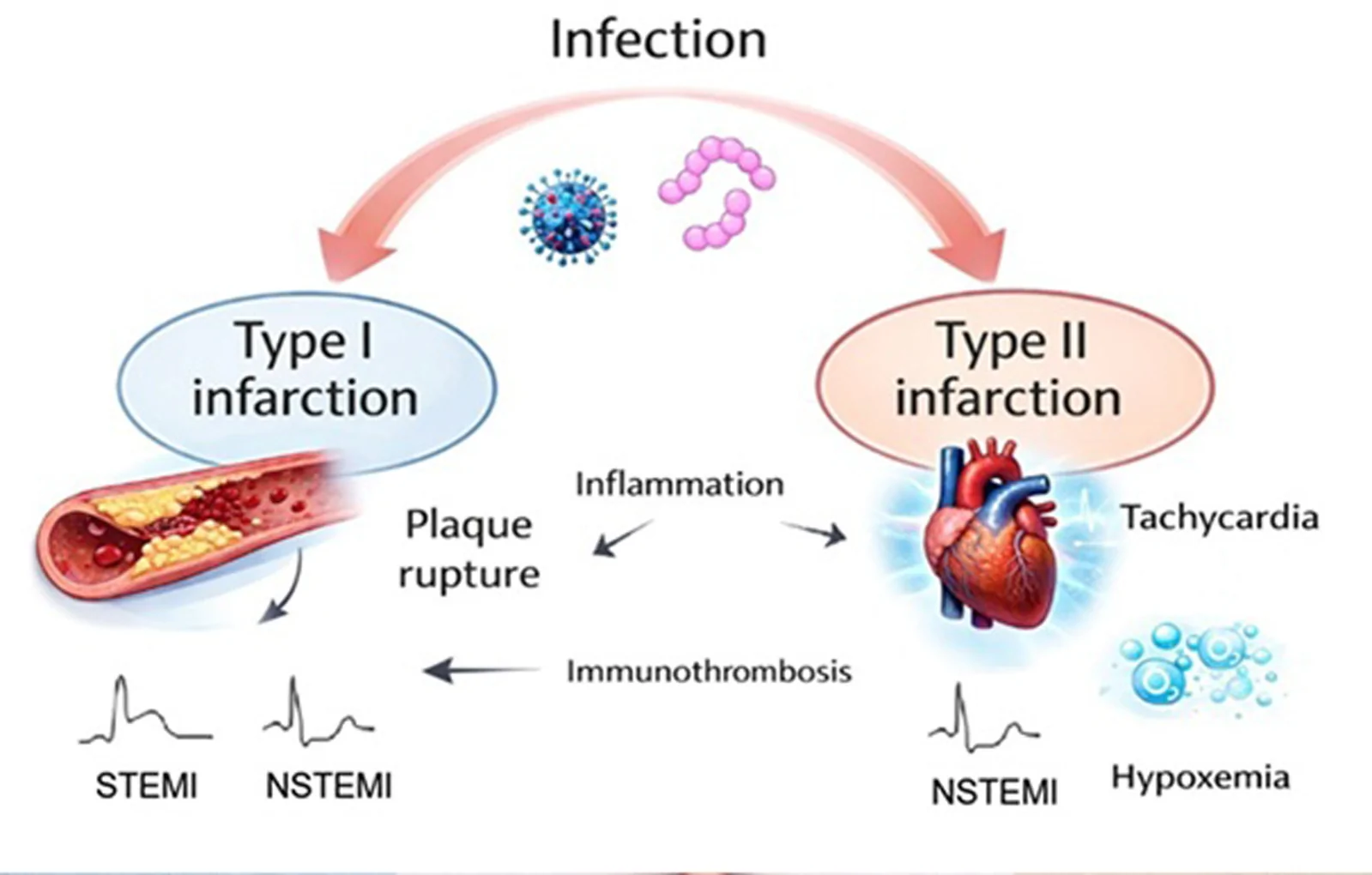
**Mechanisms by which infection can cause type I or type II myocardial infarction**. NSTEMI, non-ST-segment elevation myocardial infarction; STEMI, ST-segment elevation myocardial infarction.

The inflammatory response is key to the effect of infection on both types of myocardial infarction, although in the case of severe respiratory infections, the effect of hypoxaemia must also be taken into account. There are three mechanisms that cause an imbalance in oxygen supply: tachycardia, which increases oxygen consumption; hypoxaemia, which reduces oxygen supply; and myocardial hypoperfusion, which also reduces it. During an acute infection, tachycardia is not driven by a single cause. It reflects a combination of inflammatory response, sympathetic activation, and sometimes hypoxaemia [31]. In cases of sepsis, peripheral vasodilation is associated with hypovolaemia and hypotension, which impair myocardial perfusion, and also lead to compensatory tachycardia. The release of cytokines and endotoxins can alter endothelial function and cause microvascular dysfunction, which also results in reduced myocardial perfusion [31].

CRFs associated with endothelial dysfunction trigger the localised recruitment of inflammatory cells. This leads to the formation of lipid-rich plaques containing macrophages and T cells, which secrete cytokines such as IL-1β and IL-6 [32]. Sites where atheromatous plaques rupture are characterised by a local inflammatory process. These sites are rich in activated macrophages and T lymphocytes that strongly express HLA-DR. Local inflammation weakens the outer capsule of the plaque and facilitates its rupture by intravascular shear forces [33]. Through the activation of the innate immune response, infection produces a systemic pro-inflammatory state that increases the vulnerability of atherosclerotic plaques and enhances the thrombotic and vasoconstrictive responses, via an interaction between neutrophils, monocytes, platelets, endothelial cells, cytokines and coagulation factors [34]. Therefore, during infection, atherosclerotic plaque rupture and local thrombosis at the site of rupture are both more likely to spiral out of control, leading to vessel obstruction and myocardial necrosis.

### 2.3 The Role of Specific Infections on the Risk of Ischemic Heart Disease

Influenza is the most studied respiratory infection in terms of its association with an increased risk of cardiovascular disease and acute coronary syndrome. A case series study of 158,777 patients with laboratory-confirmed influenza via PCR between 2008 and 2019 found that the incidence of AMI was higher in patients following influenza infection than in the control group, with a relative risk of 6.16 (95% confidence interval [CI], 4.11 to 9.24) [35]. A similar study by Warren-Gash et al. [36] involving 11,208 cases of acute myocardial infarction observed a slightly lower increase in AMI risk in the first 1 to 3 days following infection (RR = 4.19 [95% CI 3.18–5.53]). This risk decreased over time, with the greatest effect observed in patients aged 80 and over (*p* = 0.023). Another study conducted in Ontario, Canada, covering the entire population registered with the public health system, yielded very similar results (IR = 6.05, 95% CI, 3.86 to 9.50). An increased risk was also observed in the elderly, influenza B infection and patients experiencing a first acute myocardial infarction. However, the analyses were insufficiently powered to identify differences within these subgroups [16]. Patients with community-acquired pneumonia are at risk of suffering a heart attack, which affects between 1% and 7% of those admitted to hospital and is more common in older people. There is also an increased risk of around 2.78–3.75 during the first 14 days after discharge that decreases with time [37]. The risk decreases gradually in the first year after infection, but remains higher than in the control group for at least ten years [15]. The persistent increase in risk observed after the first year is probably because pneumonia is more common among people at high cardiovascular risk. A study involving 206 patients with pneumonia (144 due to *S. pneumoniae* and 62 due to *H. influenzae*) found that 10.7% of patients experienced an AMI. Compared with the 1.5% rate observed among the 395 controls, the result was an odds ratio (OR) of 7.8 (95% CI 3.1–19.4) [38]. Streptococcus pneumoniae accounts for between 15% and 35% of pneumonia cases, depending on the region and the patients’ ages. Musher et al. [39] found that AMI affected 7 to 8% of patients with pneumonia caused by these bacteria. Danish health records revealed that the relative risk of experiencing a first event of AMI within 28 days of laboratory-confirmed *S. pneumoniae* infection was 20.1 during the first three days, falling to 4.9 in the second week. The risk was much greater when patients developed invasive pneumococcal pneumonia [40].

Respiratory syncytial virus (RSV) has traditionally been a major health concern for children. In adults, infection was recognised as one of many seasonal viral respiratory infections and was often grouped under the umbrella term ‘influenza-like infections’. However, the implementation of respiratory panels for multiple viruses during the pandemic has revealed the significance of this disease in adults. Together with the recent availability of vaccines against RSV, this has elevated the prominence of the infection in adult patients. Numerous studies have found that cardiovascular disease is itself a risk factor for severe RSV infection, while RSV infection increases the risk of cardiovascular complications [41]. A recent study focusing on the increased rate of cardiovascular events in elderly patients associated with RSV found a rate of 18.5% (474/2558) in patients hospitalised with RSV, compared to 17.7% (2961/16,688), 12.1% (8908/73587) and 8.4% (941/11262) in patients hospitalised with influenza, a urinary tract infection or fractures respectively. This suggests that influenza and RSV infections pose a similar risk of severe cardiovascular events [42]. The aforementioned study, which used data from the Ontario public health system registry, analysed the relationship between laboratory-confirmed RSV infection and AMI incidence, yielding an IR of 3.51 (95% CI, 1.11 to 11.12). Similarly, a cross-sectional study investigating the frequency and severity of acute cardiac events in patients with laboratory-confirmed RSV infection found a 7.5% prevalence (95% CI, 6.8–8.3) of ischemic heart disease in a sample of 6248 hospitalised patients [43]. In short, although the strength of the association may vary depending on the selection of patients in whom the virus is identified, there appears to be a clear link between RSV infection and an increased risk of an acute ischemic event.

Herpes zoster (HZ) is a neurocutaneous disease caused by the reactivation of the varicella-zoster virus, which lies dormant in the sensory ganglia. It affects around one in three people at some point in their life, and the incidence and severity of the disease increase significantly with age and a decline in immune function [44]. The varicella-zoster virus has also been linked to an increased incidence of AMI. A retrospective cohort study published in 2023 showed that patients with a documented herpes zoster infection during the study period were 1.35 times more likely to develop a myocardial infarction within 30 days of infection than those in the control group [45]. A 2017 meta-analysis also found an increased risk of myocardial infarction following HZ infection (RR: 1.18; 95% CI: 1.07–1.30) [46]. A case series study investigating whether antiviral treatment reduces the risk of AMI in patients with HZ found that the incidence of AMI per 100 person-years increased from 25.89 (95% CI: 25.43–26.35) during the reference period to 39.99 (95% CI: 33.86–47.21) within one to four weeks of HZ diagnosis (adjusted incidence risk ratio (IRR): 1.53; 95% CI: 1.29–1.82). The incidence in patients treated with antivirals was similar, with no reduction in the risk of myocardial infarction observed [47].

Coronaviruses are important pathogens for humans and animals, typically causing respiratory and gastrointestinal infections. Until 2002, they were considered minor pathogens for humans, but then an emergency occurred due to a highly pathogenic zoonotic disease: Severe Acute Respiratory Syndrome (SARS). Ten years later, a new coronavirus jumped from animals to humans, causing the lethal Middle East Respiratory Syndrome (MERS), which was first identified in humans in 2012 [48]. The 2019 pandemic wave of SARS-CoV-2, caused by COVID-19, resulted in more than 5 million confirmed deaths worldwide, with excess mortality attributable to the disease amounting to over 18 million people [49]. The severity of the respiratory syndrome and the associated immune response were accompanied by multiple systemic complications. A meta-analysis published in 2023 [18] showed that 3.5 per 1000 of patients who contracted the infection during the study period suffered an AMI, with the virus being associated with a 93% increased risk of AMI immediately following infection, which gradually decreased until it had disappeared after 8.5 months. During an average follow-up period of 8.5 months, 3.5 cases of AMI occurred per 1000 people among patients who had recovered from COVID-19. This was compared with 2.02 cases per 1000 people in the control cohort, who were defined as individuals who had not had the virus during the same period. Patients with a history of SARS-CoV-2 infection showed an increased risk of incident AMI (hazard ratio (HR): 1.93; 95% CI: 1.65–2.26). A broader cardiovascular meta-analysis revealed that the risk of myocardial infarction was approximately 4.4 times higher during the acute phase and remained elevated with RR 1.4 during the post-acute phase. The risk almost doubled for approximately one month following infection [50]. Another case series study conducted in Sweden found that excluding the day of exposure to the virus, the relative risk ratio (RRR) for AMI was 2.89 (95% CI: 1.51–5.55) in the first week, 2.53 (1.29–4.94) in the second week and 1.60 (0.84–3.04) in weeks three and four following a SARS-CoV-2 infection. When the day of exposure was included in the risk period, the IRR was 8.44 (5.45–13.08) in the first week, 2.56 (1.31–5.01) in the second week, and 1.62 (0.85–3.09) in weeks three and four following a SARS-CoV-2 infection [51]. Patients with a higher cardiovascular risk were more frequently infected and, together with patients with a history of cardiovascular disease, had a worse outcome. This has also been observed in patients with any acute lower respiratory tract infection, particularly those with RSV [43] or *S. pneumoniae*.

In short, it is important to bear in mind that respiratory infections can not only lead to adverse cardiovascular effects. They also affect those with pre-existing cardiovascular risk factors more frequently and with poorer prognosis, particularly those with established cardiovascular disease (Fig. 5). Consequently, these individuals should be a priority target group in infectious disease prevention policies.

**Fig. 5.**
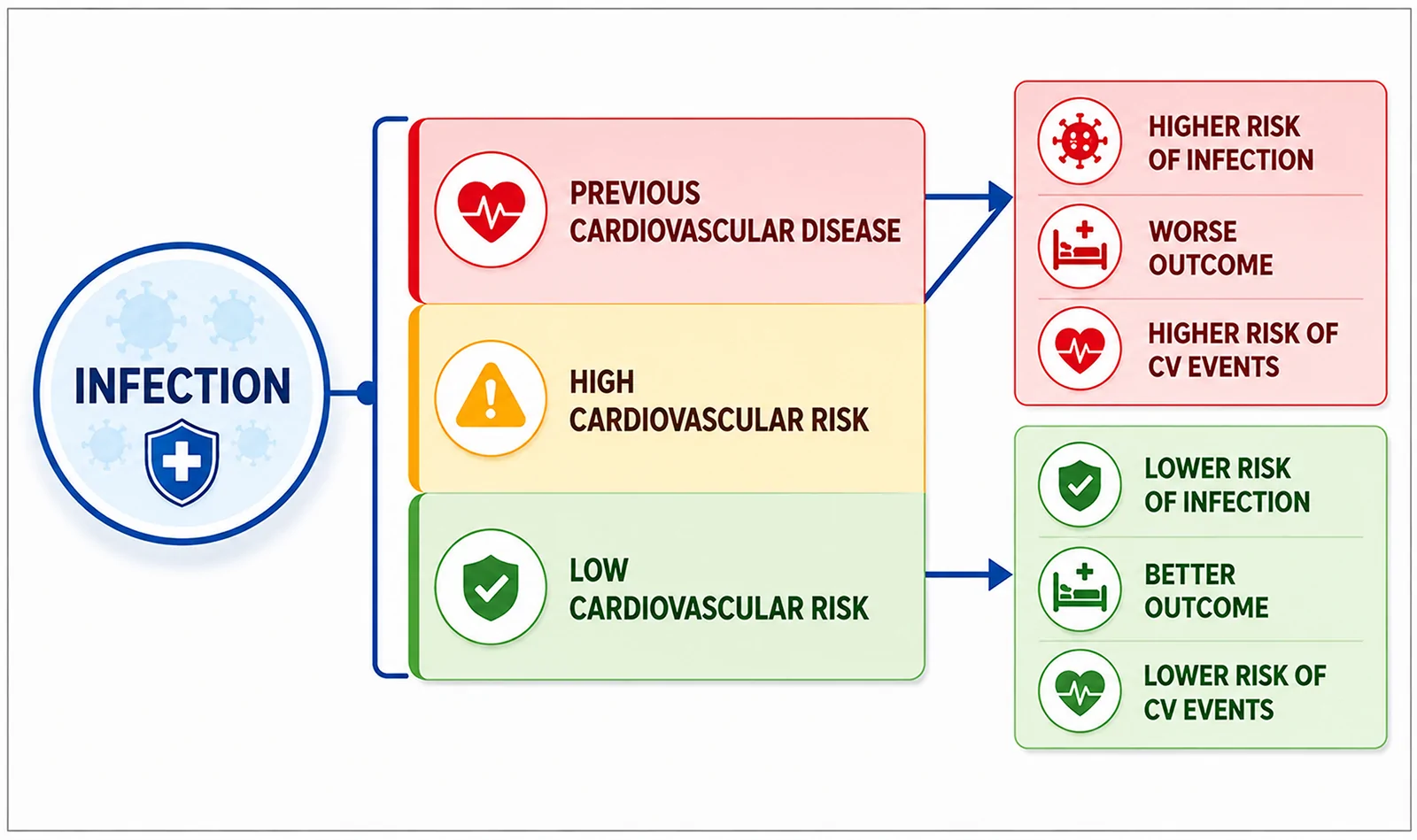
**Schematic overview of the relationship between infection and cardiovascular risk**. Not only do infections carry a higher risk of cardiovascular events, including heart attacks, they also affect people at higher cardiovascular risk more frequently. In these individuals, they are associated with a poorer prognosis and greater associated risks.

## 3. Vaccines Associated With a Reduced Risk of Acute Coronary Syndrome

### 3.1 Influenza Vaccines

Vaccines are designed to generate acquired immunity to a disease. They typically contain attenuated or inactivated forms of the causative agent, its toxins, or one of its surface proteins. Vaccines stimulate the immune system to recognise the agent as a threat, destroy it, and remember it. This enables the host to recognise and destroy the threat more easily in the event of future encounters.

The influenza virus was first isolated in 1933, leading to the development of the first monovalent vaccine using viruses grown in embryonated eggs [52]. Following the discovery of the influenza B virus, the first bivalent vaccine was developed in 1942. Since 1973, the World Health Organization (WHO) has issued annual recommendations for vaccine composition based on global surveillance systems that identify circulating strains. Trivalent vaccines containing two influenza A strains (H1N1 and H3N2) and one influenza B strain were introduced in 1978, and this has remained the standard formulation ever since [52], until recent development of tetravalent vaccines. Over time, methods of producing vaccines have evolved to encompass manufacturing in cell cultures and the use of genetic recombination techniques, ranging from attenuated viruses to specific virions and antigens. More recently, messenger ribonucleic acid (mRNA) vaccines have been developed [53,54]. This latest approach could facilitate the rapid production of vaccines tailored to circulating strains, as was seen during the SARS-CoV-2 pandemic, without the need for viral cultures in eggs. High-dose vaccines and vaccines containing adjuvants that enhance immunogenicity have also been developed to boost effectiveness. These are particularly useful for individuals with reduced immune response capacity, as elderly [53]. High-dose vaccines induce a stronger immune response than the standard vaccine. Recently, two large pragmatic clinical trials, DANFLU-2 and GALFLU, together with a pooled analysis combining both studies (FLUNITY-HD), have provided evidence on the clinical efficacy of the high-dose tetravalent vaccine compared with the standard-dose tetravalent vaccine in people aged 65 and over, including its impact on major adverse cardiovascular events (MACE) [55,56,57]. It is worth noting that both GALFLU and FLUNITY-HD showed statistically significant reductions in the primary outcome of hospital admission for influenza or pneumonia within 14 days of vaccination (unlike DANFLU-2). Sub-analyses of DANFLU-2 in patients with prior CVD, including ischemic heart disease and HF, showed no significant differences in relative effectiveness between the high-dose and standard vaccines [57]. No differences in all-cause mortality were detected, and the safety profile was similar in both groups.

Due to virus mutations and annual variation in circulating strains, the effectiveness of the flu vaccine varies from season to season. It can range from 16% to 76%, depending on the match between the strains included in the vaccine and those circulating in the population [58]. This is a key consideration when analysing studies that seek to demonstrate the protective effects of the flu vaccine on cardiovascular outcomes, as it accounts for the wide variability in results and the need to evaluate multiple vaccination campaigns. The vaccine’s effectiveness peaks one month after injection and declines over a six-month period, more rapidly in older people [58]. This is not a drawback, given the seasonal nature of the flu epidemic in countries with temperate climates. However, this justifies the fact that, alongside the seasonal changes in virus strains, vaccination should be an annual event. The campaign should begin before the period when the virus is most likely to circulate, which coincides with the start of autumn.

### 3.2 S. pneumoniae Vaccines

The first pneumococcal vaccine approved in the US was the 14-valent polysaccharide vaccine (PPV-14) in 1977. This was replaced in 1983 by a 23-valent polysaccharide vaccine (PPSV23), which is still in use today. Since 2000, conjugate vaccines have been marketed, increasing their strain coverage from seven (PCV7) to 21 (PCV21) [59]. Conjugate vaccines are an improvement on polysaccharide vaccines. They produce a more prolonged, T-cell-dependent immune response, accompanied by memory B-cell responses. They also prevent nasopharyngeal colonisation and may therefore reduce transmission and promote herd immunity [60]. Depending on the number of serotypes included, they can prevent up to 72% of invasive pneumococcal disease in adults. However, it should be noted that vaccine use itself can alter circulating strains in populations with different vaccination strategies [61]. The effectiveness of polysaccharide vaccines declines from 48% in the first two years to less than 25% after five years, but persistent antibody levels remain higher than in the unvaccinated population for six years afterwards. However, antibody persistence should not be confused with vaccine effectiveness, as some of this may depend on immune memory. Conversely, the duration of effectiveness remains stable for at least the first five years [62].

### 3.3 SARS-CoV2 Vaccines

Although coronaviruses are well-known human pathogens, the urgent need for a vaccine arose due to the COVID-19 pandemic. The first vaccines were developed in less than three months following the publication of the SARS-CoV-2 genetic sequence in January 2020, and were trialled and made available by the end of 2020. The WHO approved 10 COVID-19 vaccines representing eight distinct products across four vaccine platforms [63]: mRNA vaccines, the first to be approved for human use; adenovirus vector-based vaccines; inactivated virus vaccines; and protein subunit vaccines [63,64]. All vaccines met or exceeded the FDA’s minimum efficacy threshold of 50% protection [64], with both mRNA vaccines (BNT162b2 and mRNA-1273) providing over 90% protection against hospitalisation due to symptomatic infection [65]. During the first few years following the emergence of COVID-19, a high level of protection was observed for BNT162b2, mRNA-1273 and Ad26.COV.2.S. mRNA-1273 was particularly effective in older people and those with heart disease [66]. Recently, two new vaccines have been developed: a recombinant protein-based vaccine [67] and a new mRNA vaccine [68]. The latter is potentially the most effective in older people.

A higher incidence of myopericarditis has been reported for messenger RNA vaccines (BNT162b2 and mRNA-1273), the cause of which is not fully understood. A 2022 systematic review [69] reported 887 cases among 53 million vaccinated individuals (0.0016%), 90% of whom were young men. Twelve patients required intensive care and two died. However, the risk of myocarditis associated with SARS-CoV-2 infection was higher than that risk attributable to the vaccines. Serious thrombotic events have been reported in the case of adenoviral vector vaccines (ChAdOx1 nCoV-19 and Ad26.COV2), primarily in the cerebral venous sinuses. This is due to vaccine-induced thrombotic thrombocytopenia (VITT), which is estimated to affect 28 people per 100,000 vaccinated individuals, 70% of whom are women, with a mortality rate of 34% [70]. The risk of TT from a SARS-CoV-2 infection is estimated to be 2.3 times higher than from the vaccines. Overall, the benefits of vaccination far outweigh the potential adverse effects.

A decline in the vaccine’s efficacy has been observed during the first six months, which is more pronounced in older people [71]. This led to the consideration of a booster dose during the pandemic. Together with changes in the biology of the circulating virus, this has sparked controversy regarding the frequency of vaccination in susceptible individuals [60].

### 3.4 Respiratory Syncytial Virus Vaccines

The widespread implementation of respiratory virus screening panels during and after the SARS-CoV-2 pandemic has highlighted the significant role of other respiratory viruses, such as RSV and human metapneumovirus, in causing respiratory infection in adults. Conceptually, RSV infection has evolved from being a potentially serious infection in children to becoming a cause of hospital admission with serious consequences for older adults, especially those with comorbidities or an impaired immune system [72].

Consequently, there has recently been marked interest in developing and using vaccines to prevent infection by this seasonally occurring virus. Currently, three types of vaccine are available for adults: an adjuvanted monovalent RSV-A prefusion F protein vaccine; a bivalent RSV-A and RSV-B prefusion F protein vaccine; and an mRNA vaccine encoding stabilised prefusion F [73]. Real-world effectiveness data show 58% effectiveness against RSV-associated hospitalisation over two seasons, with 72% effectiveness against invasive mechanical ventilation or death. Protection was consistent for both RSV subtypes, A and B, at 54% and 65%, respectively. However, the vaccine was less effective in immunocompromised individuals and those with cardiovascular disease [74]. The vaccine’s protective effect is estimated to last for at least two seasons. In the case of the mRNA vaccine specifically, efficacy was shown to decline from 78.7% at a median of 3.7 months to 47.4% at a median of 18.8 months [73]. So far, no specific adverse effects have been reported for either vaccine, apart from those typically associated with reactogenicity at the injection site. The pivotal studies also do not indicate an increase in serious systemic effects [73,75].

### 3.5 Varicella-Zoster Virus Vaccine (Herpes Zoster)

The vaccine against varicella-zoster virus currently available is a recombinant vaccine (RZV), which is highly effective (over 90%) in adults over 50 years of age against the cutaneous disease and postherpetic neuralgia, which also occurs in those over 70 years of age. Its greater effectiveness and the possibility of administration in immunocompromised patients, for whom immunisation is of greater interest, have led to the live attenuated vaccine (ZVL) being ruled out [44]. Regarding its safety profile, an extremely rare association with Guillain-Barré syndrome has been reported (3–6 cases per million doses) [76]. The duration of protection may last up to 10 years, although it is considered to be at least 4 years [77]. Two doses of the RZV vaccine are recommended for adults over 50 years of age. It should be administered intramuscularly, with a 2–6-month interval between doses, regardless of whether the patient has previously had the disease or has previously received ZVL, with a minimum interval of 8 weeks. In candidates for heart transplantation, the course should be completed at least 4 weeks before the procedure [77,78].

### 3.6 Evidence Relating to the Protection Provided by Vaccines Against Ischemic Heart Disease

The increased cardiovascular risk associated with various types of infection suggests that the tools available to prevent them, particularly vaccines, may reduce cardiovascular events, particularly acute coronary syndrome, in susceptible populations. However, since vaccines activate the immune system, it is reasonable to assume that they may temporarily increase the risk of events, or that they should be avoided in unstable patients. Consequently, there has been some reluctance to vaccinate people at very high risk or during particularly vulnerable periods, such as the post-infarction period. There is extensive scientific evidence demonstrating that vaccination reduces the incidence of MACE, as well as hospitalisation and mortality rates. This benefit is particularly notable in patients with cardiovascular disease (CVD) and in other vulnerable groups [79].

Influenza vaccination has the strongest body of evidence in terms of cardiovascular benefit. Randomised clinical trials, observational studies and meta-analyses have demonstrated the effectiveness of influenza vaccination in older and/or high-risk individuals, including patients with CVD. The first randomized clinical trial to demonstrate a reduction in cardiovascular events through influenza vaccination was conducted in 2002 by Gurfinkel et al. [80]. This pilot multicenter randomized study showed a reduction of the primary endpoint, cardiovascular death, with a RR of 0.25; 95% CI 0.07 to 0.86; being this wide interval attributable to the sample size of 301 patients (200 AMI and 101 stable patients with planned angioplasty or stent). A 2013 meta-analysis of clinical trials [81] showed that influenza vaccination reduces the risk of MACE by 37% (95% CI 23–49%) in people with CVD. Similarly, a 2023 meta-analysis combining six clinical trials involving 9340 participants with a history of ischemic heart disease or heart failure found that vaccination was associated with a 26% (95% CI 12–37%) reduction in the primary composite endpoint that included cardiovascular death, acute coronary syndrome, stent thrombosis or coronary revascularization, stroke or heart failure hospitalization. The secondary endpoints were also significantly reduced, with a 37% reduction in cardiovascular mortality (95% CI 5–58%) and a 28% reduction in all-cause mortality (95% CI 5–56%) [82]. Of particular interest is the influenza vaccination after myocardial infarction (IAMI) study, which conducted a randomised, double-blind trial to compare the inactivated influenza vaccine with a saline placebo administered shortly after a myocardial infarction (99.7% of patients) or high-risk stable coronary heart disease (0.3%). The primary endpoint was the composite of all-cause death, myocardial infarction or stent thrombosis at 12 months. The study demonstrated particular benefits when the vaccine was administered early (within 72 hours of coronary angiography or admission for myocardial infarction) [83], reducing the risk of MACE, cardiovascular mortality and all-cause mortality by 28%, 41% and 41% respectively, compared with placebo. This study confirmed the safety of influenza vaccination during the acute phase following an MI.

There is clear evidence to support the use of the pneumococcal vaccine for preventing cardiovascular disease. A meta-analysis of 15 studies involving 347,000 people found a 27% reduction in the risk of AMI (HR 0.73; 95% CI 0.56–0.96), although the effect was not consistent across all components of MACE [84]. Other systematic reviews and meta-analyses demonstrate that vaccinated adults have a lower risk of developing cardiovascular disease than unvaccinated adults. However, the first prospective, randomised study to be published has failed to confirm this finding. The study included individuals aged 55–60 with no prior indication for the vaccine and at least two cardiovascular risk factors (hypertension, obesity, and/or dyslipidaemia), with no history of ischemic events. Approximately half of a sample of 4725 people with a mean age of 57 years received the 23-valent PPV vaccine. There was no reduction in the events considered in the study. However, the authors note that the study was underpowered. The sample studied was not similar in age or risk to patients who usually receive this or the current conjugate vaccines [85]. Recent population-based studies from 2024 have evaluated the effects of sequential versus single-dose vaccination in older adults on cardiovascular disease [84,86]. Evidence suggests that the single-dose regimen provides immediate and effective protection, while the sequential strategy, typically combining the conjugate and polysaccharide vaccines, aims to enhance immune memory and broaden serotype coverage. Implementing a sequential regimen has been associated with reducing long-term cardiovascular complications, thereby optimising safety and efficacy in older adults with cardiac comorbidities [86].

Vaccines to prevent respiratory syncytial virus (RSV) infection have only recently become available. There are no prospective studies on reducing cardiovascular events, and the number of observational studies is low, with smaller sample sizes. In fact, vaccination coverage for preventing RSV infection among older adults or those with cardiovascular disease remains relatively low. The DAN-RSV trial included patients aged 60 years and over who were randomly assigned to receive either the RSV-preF vaccine or no vaccine. Although a reduction in cardiorespiratory hospitalisations was demonstrated [87], a pre-specified secondary analysis showed no statistically significant reduction in MACE in participants without pre-existing atherosclerotic cardiovascular disease (9.3%, 95% CI –15.1 to 28.6) or in those with it (12.0%, 95% CI –34.6 to 43.3) [88]. Recently, TriNetX US Collaborative Network data, including around 77,000 people aged 60 or over, have shown a potential reduction in MACE (OR: 0.22, 95% CI: 0.15–0.34, *p* < 0.0001), all-cause mortality (OR: 0.16, 95% CI: 0.09–0.27, *p* < 0.0001), AMI (OR: 0.15, 95% CI: 0.08–0.31, *p* < 0.0001) and heart failure (OR: 0.32, 95% CI: 0.19–0.55, *p* < 0.0001). These differences remained significant at 6 and 12 months [89].

Patients with cardiovascular disease are more susceptible to HZ infection. A cohort study compared adults who received two doses of the recombinant zoster vaccine (RZV) with those who did not. The incidence of first-time myocardial infarction, stroke, and mortality was assessed after three years. It was observed that patients who received two doses of RZV had a lower risk of AMI (adjusted RR 0.73; 95% CI 0.55–0.96) and mortality (0.7; 95% CI 0.57–0.88), while their risk of stroke was similar (0.97; 95% CI 0.75–1.26) [90]. Studies conducted in adults aged 50 years and over have shown that the two-dose RZV regimen is associated with a reduction in cerebrovascular disorders (HR 0.76; 95% CI 0.74–0.78) and ischemic heart disease (HR 0.78; 95% CI 0.76–0.80) [91]. In patients with diabetes, vaccination is associated with HR reduction of MACE (0.76, 95% CI 0.72–0.79) and 0.54 (95% CI 0.52–0.57) HR reduction in all-cause mortality [92]. The cardioprotective effect appears to be strongest during the first year following immunisation [91,92].

It is estimated that the first year of the pandemic saw 14.4 million deaths prevented by the rollout of the COVID-19 vaccine. A study tracking 46 million people in the UK from December 2020 to January 2022 showed that vaccinated individuals were at lower risk of AMI, stroke and pulmonary thromboembolism [93]. Another study of eight million people in Sweden found that the risk of AMI, heart failure (HF), stroke and atrial fibrillation (AF) decreased from the third dose onwards. Although the first doses were associated with an increased risk of myopericarditis and extrasystoles [94], the authors concluded that the reduction in events due to the vaccine outweighed the described complications. Other observational studies involving large sample sizes and various types of vaccines have produced similar results [95,96]. However, there are no prospective randomised trials, and the available data are from periods close to the critical phase of the pandemic. Until more recent evidence becomes available, the existing data support the idea that vaccines developed against the SARS-CoV-2 virus reduce the risk of cardiovascular events.

## 4. Vaccination to Prevent Cardiac Events in the Guidelines

To the best of our knowledge, the 2020 consensus statement on immunisation in adults with cardiovascular disease issued by the Argentine Society of Cardiology is the first to refer to vaccination of people with cardiovascular disease as a tool for secondary prevention, with a level of evidence I for many of its recommendations [97]. This consensus document provides a detailed analysis of vaccination requirements, including, for example, how to administer intramuscular vaccines to patients receiving anticoagulant therapy. The guidelines of the European Society of Cardiology (ESC) for the management of heart failure included influenza and pneumococcal vaccination for the first time as a means of reducing hospitalisations for heart failure in 2021 [98], with a Class 2 recommendation (should be considered). In the same year, the ESC cardiovascular prevention guidelines made no recommendation on this matter, placing vaccination on the same level as periodontal infection, devoting only 38 words to this issue [99]. In 2022, the guidelines of the American Heart Association (AHA) and the American College of Cardiology (ACC) for the management of heart failure recommended vaccination against “respiratory infections” to reduce mortality from heart failure, also with a level of evidence 2a [100]. In 2023 the flu vaccine was listed as a Class IA indication (strong recommendation) to reduce overall and cardiovascular mortality, and to reduce cardiovascular morbidity in patients with chronic coronary artery disease. With a level of evidence of IC, they recommended vaccination against COVID “in accordance with public health guidelines”, whilst vaccination against pneumococcus was listed at level 2b as a reasonable proposal to reduce all-cause and cardiovascular mortality, and to reduce cardiovascular morbidity in patients with chronic coronary artery disease [101]. The 2023 update of the European guidelines on heart failure did not include any changes compared to those of 2021 [102].

In 2025, the European Society of Cardiology issued a consensus document entitled “Vaccination as a new form of cardiovascular prevention: a European Society of Cardiology clinical consensus statement” reviewing multiple vaccines and clinical situations. As a consensus document, levels of recommendation were not established [103]. For their part, the AHA and the ACC issued a consensus document in the same year (Expert Consensus Statement on Adult Immunizations as Part of Cardiovascular Care) [60] in which, also without establishing levels of evidence, they recommend vaccination for people with cardiovascular disease against influenza, pneumococcal disease, COVID-19, respiratory syncytial virus and herpes zoster. Four reasons for this are stated: the poorer prognosis of patients with cardiovascular disease when they contract an infection; the ability of approved vaccines to reduce severe infection; data from observational studies; and the rarity of serious adverse effects from the vaccines. The recommendations of both statements are condensed in Table 1 (Ref. 52,53,58,59,60,63,64,65,66,67,68,69,70,71,72,73,74,76,77,83,103,104,105,106,107). Similar approaches have been taken by other cardiology societies in different countries and regions, such as the Canadian, Polish and Argentine ones [104,105,106]. This shift in recommendations suggests that cardiologists’ views on vaccination are aligning with those of public health experts. It is a matter of enormous importance, given that despite public campaigns promoting various forms of vaccination, most countries are far from achieving adequate vaccination rates. Just as an example, during the 2021–22 season, the average flu vaccination rate among those aged 65 and older in European countries, the U.S., and Canada was only 48.1%, far below the 75% recommended by the World Health Organization, and only 5 of 31 countries reaching the objective [107]. Recognizing that vaccines are a key tool in cardiovascular prevention should make cardiologists allies of vaccination campaigns, helping to ensure that these campaigns reach particularly vulnerable groups.

**Table 1.** **Summary of recommendations for the vaccination of patients with a history of coronary heart disease (adapted from the recommendations of North American, European and Argentine societies of cardiology) [60,103,104,105,106]**.

Vaccine	Seasonal	Intervals	Specific recommendations and comments
Influenza [52,53,58]	Yes	Yearly	Preferably before discharge in patients hospitalized for acute coronary syndrome [83]
*S. pneumoniae* [59,60,72]	Yes	5–10 years^1^	Better associated to Influenza vaccine. If available, use conjugate PCV20 or PCV21 If not, a sequential schedule with PCV13 followed by PPSV23 could be advisable
COVID-19 [63,64,65,66,67,68,69,70,71]	Yes^2^	Yearly^2^	Best administered alongside the influenza vaccine to improve adherence [107]
RSV [73,74]	Yes	Not defined	The protection provided by RSV vaccines could last longer than three years
Herpes Zoster [76,77]	No	2 doses series once in life	Standard two-dose schedule of recombinant vaccine (RZV) separated 2 to 6 months could last 10–11 years

^1^ The effect could last longer, but it could be necessary to re-vaccinate specific populations. ^2^ COVID-19 could not be a seasonal epidemic, and the effect of the vaccine lasts less than a year. The need to vaccinate every six months could be discussed. Due to public health policies, it is advisable to administer the vaccine yearly, together with the influenza vaccine [60,71]. RSV, respiratory syncytial virus.

## 5. Conclusions

Vaccines reduce the risk of many infectious diseases and their complications, which use to be more frequent in patients with a high cardiovascular risk and a history of cardiovascular disease. There is compelling evidence linking infections to an increased risk of cardiac ischemic events. While the evidence supporting a reduction in the risk of a myocardial infarction through vaccination is less extensive, it is nevertheless consistent, particularly with regard to influenza vaccination. Importantly, vaccination is safe for patients who have experienced a recent cardiovascular event.

Cardiologists and other healthcare professionals should regard vaccination recommendations as equally important as advice on diet, physical exercise and giving up smoking. Furthermore, they should view their own vaccination as a means of protecting their patients; this responsible attitude should also help them to remember the need to recommend it. The prescription of vaccines for adults should form part of standard prescribing practice, alongside other therapies aimed at reducing cardiovascular risk.

## Abbreviations

ACC, American College of Cardiology; ACS, acute coronary syndrome; AHA, American Heart Association; AMI, acute myocardial infarction; CI, confidence interval; CMV, cytomegalovirus; CRFs, cardiovascular risk factors; ESC, European Society of Cardiology; HR, hazard ratio; HZ, herpes zoster; HIV, human immunodeficiency virus; IHD, ischemic heart disease; ILI, influenza like infections; MACE, major adverse cardiovascular events; NSTEMI, non-ST-segment elevation myocardial infarction; RR, risk ratio; RSV, respiratory syncytial virus; SARS, severe acute respiratory syndrome; STEMI, ST-segment elevation myocardial infarction; VITT, vaccine induced thrombotic thrombocytopenia.

## References

[1] GBD 2023 Causes of Death Collaborators (2025). Global burden of 292 causes of death in 204 countries and territories and 660 subnational locations, 1990-2023: a systematic analysis for the Global Burden of Disease Study 2023. Lancet (London, England).

[2] Díaz-Polanco JC, Tejada-González C, Leandro-Barros A, Ruiz-Saavedra A, García-de-Santiago E, Alonso-Martín J (2024). Seasonal Stability of the Circadian Rhythm in Patients with Type I Myocardial Infarction. Reviews in Cardiovascular Medicine.

[3] Fowler T, Southgate RJ, Waite T, Harrell R, Kovats S, Bone A (2015). Excess winter deaths in Europe: a multi-country descriptive analysis. European Journal of Public Health.

[4] Carlos III Health Institute MoMo: Daily Mortality Monitoring System. Madrid: ISCIII. https://momo.isciii.es/panel_momo/.

[5] García-Lledó A, Rodríguez-Martín S, Tobías A, García-de-Santiago E, Ordobás-Gavín M, Ansede-Cascudo JC (2021). Relationship Between Influenza, Temperature, and Type 1 Myocardial Infarction: An Ecological Time-Series Study. Journal of the American Heart Association.

[6] Gong XY, Dong ZC, Sha TT, Li Q, Dou YJ, Zhang HX (2026). Interactive effects of diurnal temperature range and air pollution on myocardial infarction incidence: an individual-level case-crossover study. Scientific Reports.

[7] García-de-Santiago E, Lozano-Batuecas M, García-Pérez-Velasco J, Gómez-Delgado J, García-Arribas D, Herruzo-León A (2025). Wealth, Unemployment, Social Investment, and Risk of ST-Segment Elevation Myocardial Infarction-An Ecological Analysis of a Low-Cardiovascular-Risk European Region. Journal of Clinical Medicine.

[8] Bergen N, Kirkby K, Fuertes CV, Schlotheuber A, Menning L, Mac Feely S (2023). Global state of education-related inequality in COVID-19 vaccine coverage, structural barriers, vaccine hesitancy, and vaccine refusal: findings from the Global COVID-19 Trends and Impact Survey. The Lancet. Global Health.

[9] Hanneman K, Alahmad B, Ghosh A, Khatana SAM, Huang M, Liu J (2026). Nonoptimal Temperature and Cardiovascular Health: A Scientific Statement From the American Heart Association. Circulation.

[10] Smeeth L, Thomas SL, Hall AJ, Hubbard R, Farrington P, Vallance P (2004). Risk of myocardial infarction and stroke after acute infection or vaccination. The New England Journal of Medicine.

[11] Pedersen EMJ, Yonis H, Egelund GB, Lohse N, Torp-Pedersen C, Lindegaard B (2025). Severe infections as risk factors for acute myocardial infarction: a nationwide, Danish cohort study from 1987 to 2018. European Journal of Preventive Cardiology.

[12] Corrales-Medina VF, Fatemi O, Serpa J, Valayam J, Bozkurt B, Madjid M (2009). The association between Staphylococcus aureus bacteremia and acute myocardial infarction. Scandinavian Journal of Infectious Diseases.

[13] Kawai K, Muhere CF, Lemos EV, Francis JM (2025). Viral Infections and Risk of Cardiovascular Disease: Systematic Review and Meta-Analysis. Journal of the American Heart Association.

[14] Wee LE, Lim JT, Ho RWL, Chiew CJ, Lye DCB, Tan KB (2025). Cardiac Events in Adults Hospitalized for Respiratory Syncytial Virus vs COVID-19 or Influenza. JAMA Network Open.

[15] Corrales-Medina VF, Alvarez KN, Weissfeld LA, Angus DC, Chirinos JA, Chang CCH (2015). Association between hospitalization for pneumonia and subsequent risk of cardiovascular disease. JAMA.

[16] Kwong JC, Schwartz KL, Campitelli MA, Chung H, Crowcroft NS, Karnauchow T (2018). Acute Myocardial Infarction after Laboratory-Confirmed Influenza Infection. The New England Journal of Medicine.

[17] Warren-Gash C, Blackburn R, Whitaker H, McMenamin J, Hayward AC (2018). Laboratory-confirmed respiratory infections as triggers for acute myocardial infarction and stroke: a self-controlled case series analysis of national linked datasets from Scotland. The European Respiratory Journal.

[18] Zuin M, Rigatelli G, Battisti V, Costola G, Roncon L, Bilato C (2023). Increased risk of acute myocardial infarction after COVID-19 recovery: A systematic review and meta-analysis. International Journal of Cardiology.

[19] Warren-Gash C, Davidson JA, Strongman H, Herrett E, Smeeth L, Breuer J (2023). Severe COVID-19 outcomes by cardiovascular risk profile in England in 2020: a population-based cohort study. The Lancet Regional Health. Europe.

[20] Kaklikkaya I, Kaklikkaya N, Birincioglu I, Buruk K, Turan N (2010). Detection of human herpesvirus 6 DNA but not human herpesvirus 7 or 8 DNA in atherosclerotic and nonatherosclerotic vascular tissues. The Heart Surgery Forum.

[21] Šačić D, Tomić U, Milašin J, Putnik S, Jovanović M, Radojević Škodrić S (2024). The Detection of *Chlamydia pneumoniae*, *Helicobacter pylori* and *Cytomegalovirus* in Non-Atherosclerotic Arteries of Patients with Coronary Artery Disease. Pathogens (Basel, Switzerland).

[22] Hetta HF, Alanazi FE, Alshareef H, Alqifari SF, Bukhari SQ, Albalwi MA (2026). Antiviral Drugs in HIV and Cardiovascular Disease: Mechanistic Insights and Clinical Implications. Pharmaceuticals (Basel, Switzerland).

[23] Zheng J, Yang Q, Huang J, Chen H, Shen J, Tang S (2024). Hospital-treated infectious diseases, genetic susceptibility and risk of type 2 diabetes: A population-based longitudinal study. Diabetes & Metabolic Syndrome.

[24] Bomfim GF, Priviero F, Poole E, Tostes RC, Sinclair JH, Stamou D (2023). Cytomegalovirus and Cardiovascular Disease: A Hypothetical Role for Viral G-Protein-Coupled Receptors in Hypertension. American Journal of Hypertension.

[25] Gaonkar A, Zahiruddin QS, Shabil M, Menon SV, Kaur M, Kumari M (2025). Association of *Helicobacter pylori* Infection and Risk of Dyslipidemia: A Systematic Review and Meta-Analysis. JGH Open: an Open Access Journal of Gastroenterology and Hepatology.

[26] Thygesen K, Alpert JS, Jaffe AS, Chaitman BR, Bax JJ, Morrow DA (2019). Fourth universal definition of myocardial infarction (2018). European Heart Journal.

[27] Byrne RA, Rossello X, Coughlan JJ, Barbato E, Berry C, Chieffo A (2023). 2023 ESC Guidelines for the management of acute coronary syndromes. European Heart Journal.

[28] Roston TM, Aghanya V, Savu A, Fordyce CB, Lawler PR, Jentzer J (2024). Premature Acute Myocardial Infarction Treated With Invasive Revascularization: Comparing STEMI With NSTEMI in a Population-Based Study of Young Patients. The Canadian Journal of Cardiology.

[29] Vejpongsa P, Kitkungvan D, Madjid M, Charitakis K, Anderson HV, Arain S (2019). Outcomes of Acute Myocardial Infarction in Patients with Influenza and Other Viral Respiratory Infections. The American Journal of Medicine.

[30] Harris JE, Shah PJ, Korimilli V, Win H (2019). Frequency of troponin elevations in patients with influenza infection during the 2017-2018 influenza season. International Journal of Cardiology.

[31] Hayase N, Yamamoto M, Asada T, Isshiki R, Doi K (2024). Tachycardia and Acute Kidney Injury among Critically Ill Patients with Sepsis: A Prospective Observational Study. Blood Purification.

[32] Libby P (2021). Inflammation during the life cycle of the atherosclerotic plaque. Cardiovascular Research.

[33] van der Wal AC, Becker AE, van der Loos CM, Das PK (1994). Site of intimal rupture or erosion of thrombosed coronary atherosclerotic plaques is characterized by an inflammatory process irrespective of the dominant plaque morphology. Circulation.

[34] Stark K, Massberg S (2021). Interplay between inflammation and thrombosis in cardiovascular pathology. Nature Reviews. Cardiology.

[35] de Boer AR, Riezebos-Brilman A, van Hout D, van Mourik MSM, Rümke LW, de Hoog MLA (2024). Influenza Infection and Acute Myocardial Infarction. NEJM Evidence.

[36] Warren-Gash C, Hayward AC, Hemingway H, Denaxas S, Thomas SL, Timmis AD (2012). Influenza infection and risk of acute myocardial infarction in England and Wales: a CALIBER self-controlled case series study. The Journal of Infectious Diseases.

[37] Carneiro J, Leite A, Lahuerta M, Catusse J, Ali M, Teixeira R (2025). Risk of stroke or myocardial infarction hospitalisation following hospitalisation for community-acquired pneumonia in Portugal: a self-controlled case series study. BMJ Open.

[38] Corrales-Medina VF, Serpa J, Rueda AM, Giordano TP, Bozkurt B, Madjid M (2009). Acute bacterial pneumonia is associated with the occurrence of acute coronary syndromes. Medicine.

[39] Musher DM, Rueda AM, Kaka AS, Mapara SM (2007). The association between pneumococcal pneumonia and acute cardiac events. Clinical Infectious Diseases: an Official Publication of the Infectious Diseases Society of America.

[40] Ohland J, Warren-Gash C, Blackburn R, Mølbak K, Valentiner-Branth P, Nielsen J (2020). Acute myocardial infarctions and stroke triggered by laboratory-confirmed respiratory infections in Denmark, 2010 to 2016. European Communicable Disease Bulletin.

[41] Penders Y, Falsey AR, Bhatt DL, Böhm M, Coats AJS, Mason LMK (2025). Burden and severity of respiratory syncytial virus infection in adults with cardiovascular diseases: A systematic literature review. Progress in Cardiovascular Diseases.

[42] Verschoor CP, Cerasuolo JO, Caswell JM, Tatangelo M, Costa-Vitali A, Savage DW (2025). Respiratory Syncytial Virus (RSV)-Related Hospitalization and Increased Rate of Cardiovascular Events in Older Adults. Journal of the American Geriatrics Society.

[43] Woodruff RC, Melgar M, Pham H, Sperling LS, Loustalot F, Kirley PD (2024). Acute Cardiac Events in Hospitalized Older Adults With Respiratory Syncytial Virus Infection. JAMA Internal Medicine.

[44] de Oliveira Gomes J, Gagliardi AM, Andriolo BN, Torloni MR, Andriolo RB, Puga MEDS (2023). Vaccines for preventing herpes zoster in older adults. The Cochrane Database of Systematic Reviews.

[45] Parameswaran GI, Drye AF, Wattengel BA, Carter MT, Doyle KM, Mergenhagen KA (2023). Increased Myocardial Infarction Risk Following Herpes Zoster Infection. Open Forum Infectious Diseases.

[46] Zhang Y, Luo G, Huang Y, Yu Q, Wang L, Li K (2017). Risk of Stroke/Transient Ischemic Attack or Myocardial Infarction with Herpes Zoster: A Systematic Review and Meta-Analysis. Journal of Stroke and Cerebrovascular Diseases: the Official Journal of National Stroke Association.

[47] Lai HY, Meng LC, Chen HM, Chuang HM, Chen LK, Hsiao FY (2026). Herpes zoster infection and myocardial infarction risk: Evaluating the impact of antiviral therapy in a nationwide self-controlled case series study. Journal of Infection and Public Health.

[48] Salata C, Calistri A, Parolin C, Palù G (2019). Coronaviruses: a paradigm of new emerging zoonotic diseases. Pathogens and Disease.

[49] COVID-19 Excess Mortality Collaborators (2022). Estimating excess mortality due to the COVID-19 pandemic: a systematic analysis of COVID-19-related mortality, 2020-21. Lancet (London, England).

[50] Romero Starke K, Kaboth P, Rath N, Reissig D, Kaempf D, Nienhaus A (2024). Cardiovascular disease risk after a SARS-CoV-2 infection: A systematic review and meta-analysis. The Journal of Infection.

[51] Katsoularis I, Fonseca-Rodríguez O, Farrington P, Lindmark K, Fors Connolly AM (2021). Risk of acute myocardial infarction and ischaemic stroke following COVID-19 in Sweden: a self-controlled case series and matched cohort study. Lancet (London, England).

[52] Kim YH, Hong KJ, Kim H, Nam JH (2022). Influenza vaccines: Past, present, and future. Reviews in Medical Virology.

[53] Hsu D, Jayaraman A, Pucci A, Joshi R, Mancini K, Chen HL (2025). Safety and immunogenicity of mRNA-based seasonal influenza vaccines formulated to include multiple A/H3N2 strains with or without the B/Yamagata strain in US adults aged 50-75 years: a phase 1/2, open-label, randomised trial. The Lancet. Infectious Diseases.

[54] Fitz-Patrick D, McVinnie DS, Jackson LA, Crowther G, Geevarughese A, Cannon KD (2025). Efficacy, Immunogenicity, and Safety of Modified mRNA Influenza Vaccine. The New England Journal of Medicine.

[55] Johansen ND, Modin D, Loiacono MM, Harris RC, Dufournet M, Larsen CS (2025). High-Dose Influenza Vaccine Effectiveness against Hospitalization in Older Adults. The New England Journal of Medicine.

[56] Johansen ND, Modin D, Pardo-Seco J, Rodriguez-Tenreiro-Sánchez C, Loiacono MM, Harris RC (2025). Effectiveness of high-dose influenza vaccine against hospitalisations in older adults (FLUNITY-HD): an individual-level pooled analysis. Lancet (London, England).

[57] Johansen ND, Modin D, Loiacono MM, Harris RC, Dufournet M, Larsen CS (2025). High-Dose vs Standard-Dose Influenza Vaccine and Cardiovascular Outcomes in Older Adults: A Prespecified Secondary Analysis of the DANFLU-2 Randomized Clinical Trial. JAMA Cardiology.

[58] Osterholm MT, Kelley NS, Sommer A, Belongia EA (2012). Efficacy and effectiveness of influenza vaccines: a systematic review and meta-analysis. The Lancet. Infectious Diseases.

[59] Mirsaeidi M, Schraufnagel DE (2014). Pneumococcal vaccines: understanding centers for disease control and prevention recommendations. Annals of the American Thoracic Society.

[60] A Heidenreich P, Bhatt A, Nazir NT, Schaffner W, Vardeny O (2025). 2025 Concise Clinical Guidance: An ACC Expert Consensus Statement on Adult Immunizations as Part of Cardiovascular Care: A Report of the American College of Cardiology Solution Set Oversight Committee. Journal of the American College of Cardiology.

[61] Garcia Quesada M, Peterson ME, Bennett JC, Hayford K, Zeger SL, Yang Y (2025). Serotype distribution of remaining invasive pneumococcal disease after extensive use of ten-valent and 13-valent pneumococcal conjugate vaccines (the PSERENADE project): a global surveillance analysis. The Lancet. Infectious Diseases.

[62] Huang L, Yang X, Li H, Xie Z, Zhu T, You W (2025). Immune persistence of a single dose of 23-valent pneumococcal polysaccharide vaccine: A 6-year follow-up. Human Vaccines & Immunotherapeutics.

[63] Barouch DH (2022). Covid-19 Vaccines - Immunity, Variants, Boosters. The New England Journal of Medicine.

[64] Poland GA, Ovsyannikova IG, Kennedy RB (2020). SARS-CoV-2 immunity: review and applications to phase 3 vaccine candidates. Lancet (London, England).

[65] Baden LR, El Sahly HM, Essink B, Kotloff K, Frey S, Novak R (2021). Efficacy and Safety of the mRNA-1273 SARS-CoV-2 Vaccine. The New England Journal of Medicine.

[66] Winkelman TNA, Rai NK, Bodurtha PJ, Chamberlain AM, DeSilva M, Jeruzal J (2022). Trends in COVID-19 Vaccine Administration and Effectiveness Through October 2021. JAMA Network Open.

[67] Alves K, Kouassi A, Nelson J, Plested JS, Kalkeri R, Zhu M (2026). Safety and immunogenicity of a single dose of a JN.1 variant COVID-19 vaccine in previously vaccinated adults: Primary analysis report of a phase 3 open-label trial. Vaccine.

[68] Chalkias S, Dennis P, Petersen D, Radhakrishnan K, Vaughan L, Handforth R (2025). Efficacy, immunogenicity, and safety of a next-generation mRNA-1283 COVID-19 vaccine compared with the mRNA-1273 vaccine (NextCOVE): results from a phase 3, randomised, observer-blind, active-controlled trial. The Lancet. Infectious Diseases.

[69] Rout A, Suri S, Vorla M, Kalra DK (2022). Myocarditis associated with COVID-19 and its vaccines - a systematic review. Progress in Cardiovascular Diseases.

[70] Ohaeri C, Thomas DR, Salmon J, Cottrell S, Lyons J, Akbari A (2022). Comparative risk of cerebral venous sinus thrombosis (CVST) following COVID-19 vaccination or infection: A national cohort study using linked electronic health records. Human Vaccines & Immunotherapeutics.

[71] Feikin DR, Higdon MM, Abu-Raddad LJ, Andrews N, Araos R, Goldberg Y (2022). Duration of effectiveness of vaccines against SARS-CoV-2 infection and COVID-19 disease: results of a systematic review and meta-regression. Lancet (London, England).

[72] Havers FP, Whitaker M, Melgar M, Pham H, Chai SJ, Austin E (2024). Burden of Respiratory Syncytial Virus-Associated Hospitalizations in US Adults, October 2016 to September 2023. JAMA Network Open.

[73] Wilson E, Goswami J, Baqui AH, Doreski PA, Perez-Marc G, Zaman K (2023). Efficacy and Safety of an mRNA-Based RSV PreF Vaccine in Older Adults. The New England Journal of Medicine.

[74] Surie D, Self WH, Yuengling KA, Lauring AS, Zhu Y, Safdar B (2025). RSV Vaccine Effectiveness Against Hospitalization Among US Adults Aged 60 Years or Older During 2 Seasons. JAMA.

[75] Falsey AR, Williams K, Gymnopoulou E, Bart S, Ervin J, Bastian AR (2023). Efficacy and Safety of an Ad26.RSV.preF-RSV preF Protein Vaccine in Older Adults. The New England Journal of Medicine.

[76] Mbinta JF, Wang AX, Nguyen BP, Paynter J, Awuni PMA, Pine R (2023). Herpes zoster vaccine safety in the Aotearoa New Zealand population: a self-controlled case series study. Nature Communications.

[77] Tseng HF, Sy LS, Ackerson BK, Rayens E, Wu J, Luo Y (2026). Effectiveness of the Adjuvanted Recombinant Zoster Vaccine in Adults ≥50 Years in the United States. Clinical Infectious Diseases: an Official Publication of the Infectious Diseases Society of America.

[78] Miller G, Schaefer H, Yoder S, Miller R, Winokur P, Kotloff K (2018). A randomized, placebo-controlled phase I trial of live, attenuated herpes zoster vaccine in subjects with end-stage renal disease immunized prior to renal transplantation. Transplant Infectious Disease.

[79] Nguyen TQ, Vlasenko D, Shetty AN, Zhao E, Reid CM, Clothier HJ (2025). Systematic review and meta-analysis of respiratory viral triggers for acute myocardial infarction and stroke. Cardiovascular Research.

[80] Gurfinkel EP, de la Fuente RL, Mendiz O, Mautner B (2002). Influenza vaccine pilot study in acute coronary syndromes and planned percutaneous coronary interventions: the FLU Vaccination Acute Coronary Syndromes (FLUVACS) Study. Circulation.

[81] Udell JA, Zawi R, Bhatt DL, Keshtkar-Jahromi M, Gaughran F, Phrommintikul A (2013). Association between influenza vaccination and cardiovascular outcomes in high-risk patients: a meta-analysis. JAMA.

[82] Modin D, Lassen MCH, Claggett B, Johansen ND, Keshtkar-Jahromi M, Skaarup KG (2023). Influenza vaccination and cardiovascular events in patients with ischaemic heart disease and heart failure: A meta-analysis. European Journal of Heart Failure.

[83] Fröbert O, Götberg M, Erlinge D, Akhtar Z, Christiansen EH, MacIntyre CR (2021). Influenza Vaccination After Myocardial Infarction: A Randomized, Double-Blind, Placebo-Controlled, Multicenter Trial. Circulation.

[84] Jaiswal V, Ang SP, Lnu K, Ishak A, Pokhrel NB, Chia JE (2022). Effect of Pneumococcal Vaccine on Mortality and Cardiovascular Outcomes: A Systematic Review and Meta-Analysis. Journal of Clinical Medicine.

[85] Hure A, Peel R, D'Este C, Abhayaratna WP, Tonkin A, Hopper I (2025). Prevention of Adverse Cardiovascular Events Using the 23-Valent Pneumococcal Polysaccharide Vaccine: A Randomized Clinical Trial. JAMA Cardiology.

[86] Tong X, Gao L, Wong ICK, Chan VKY, Wong AYS, Mak JCW (2024). Effects of sequential vs single pneumococcal vaccination on cardiovascular diseases among older adults: a population-based cohort study. International Journal of Epidemiology.

[87] Lassen MCH, Johansen ND, Christensen SH, Aliabadi N, Skaarup KG, Modin D (2025). Bivalent RSV Prefusion F Protein-Based Vaccine for Preventing Cardiovascular Hospitalizations in Older Adults: A Prespecified Analysis of the DAN-RSV Trial. JAMA.

[88] Pareek M, Lassen MCH, Johansen ND, Christensen SH, Aliabadi N, Skaarup KG (2025). Effectiveness of bivalent respiratory syncytial virus prefusion F protein-based vaccine in individuals with or without atherosclerotic cardiovascular disease: the DAN-RSV trial. European Heart Journal.

[89] Arunachalam S, Anuforo A, Hetelekides S, Olojakpoke E, Gupta N, Pichs Diez A (2025). Real-World Cardiorespiratory Impact of Respiratory Syncytial Virus (RSV) Vaccination in the USA: A Retrospective Propensity-Matched Analysis. Circulation.

[90] Helm MF, Khoury PA, Pakchanian H, Raiker R, Maczuga S, Foulke GT (2023). Recombinant Zoster Vaccine Reduces 3-Year Cardiovascular Risk: Insights From a Multi-Centered Database. Journal of Drugs in Dermatology: JDD.

[91] Lee S, Lee K, Oh J, Kim HJ, Son Y, Kim S (2025). Live zoster vaccination and cardiovascular outcomes: a nationwide, South Korean study. European Heart Journal.

[92] Kornelius E, Lo SC, Huang CN, Wang CC, Wang YH, Yang YS (2025). Association of herpes zoster vaccination and cardiovascular risk in patients with diabetes: long-term insights from a retrospective cohort study. BMJ Open.

[93] Ip S, North TL, Torabi F, Li Y, Abbasizanjani H, Akbari A (2024). Cohort study of cardiovascular safety of different COVID-19 vaccination doses among 46 million adults in England. Nature Communications.

[94] Xu Y, Li H, Santosa A, Wettermark B, Fall T, Björk J (2025). Cardiovascular events following coronavirus disease 2019 vaccination in adults: a nationwide Swedish study. European Heart Journal.

[95] Akbar UA, Thyagaturu H, Taha A, Atti L, Khan A, Raina S (2025). COVID-19 Vaccination and Cardiovascular Outcomes in Older Adults With Coronary Artery Disease and Heart Failure: Insights From a Large Propensity-Matched Cohort Study. Journal of the American Heart Association.

[96] Mercadé-Besora N, Li X, Kolde R, Trinh NT, Sanchez-Santos MT, Man WY (2024). The role of COVID-19 vaccines in preventing post-COVID-19 thromboembolic and cardiovascular complications. Heart (British Cardiac Society).

[97] García-Zamora S, Sosa Liprandi MI, Picco JM, Matta MG, Villarreal R, Pulido L (2020). Inmunizaciones en adultos con cardiopatías. Resumen del Consenso de la Sociedad Argentina de Cardiología [Immunizations in adults with cardiovascular disease. Summary of the Consensus of the Argentine Cardiology Society]. MEDICINA (Buenos Aires).

[98] McDonagh TA, Metra M, Adamo M, Gardner RS, Baumbach A, Böhm M (2021). 2021 ESC Guidelines for the diagnosis and treatment of acute and chronic heart failure. European Heart Journal.

[99] Visseren FLJ, Mach F, Smulders YM, Carballo D, Koskinas KC, Bäck M (2021). 2021 ESC Guidelines on cardiovascular disease prevention in clinical practice. European Heart Journal.

[100] Heidenreich PA, Bozkurt B, Aguilar D, Allen LA, Byun JJ, Colvin MM (2022). 2022 AHA/ACC/HFSA Guideline for the Management of Heart Failure: A Report of the American College of Cardiology/American Heart Association Joint Committee on Clinical Practice Guidelines. Journal of the American College of Cardiology.

[101] Virani SS, Newby LK, Arnold SV, Bittner V, Brewer LC, Demeter SH (2023). 2023 AHA/ACC/ACCP/ASPC/NLA/PCNA Guideline for the Management of Patients With Chronic Coronary Disease: A Report of the American Heart Association/American College of Cardiology Joint Committee on Clinical Practice Guidelines. Circulation.

[102] McDonagh TA, Metra M, Adamo M, Gardner RS, Baumbach A, Böhm M (2024). 2023 Focused Update of the 2021 ESC Guidelines for the diagnosis and treatment of acute and chronic heart failure: Developed by the task force for the diagnosis and treatment of acute and chronic heart failure of the European Society of Cardiology (ESC) With the special contribution of the Heart Failure Association (HFA) of the ESC. European Journal of Heart Failure.

[103] Heidecker B, Libby P, Vassiliou VS, Roubille F, Vardeny O, Hassager C (2025). Vaccination as a new form of cardiovascular prevention: a European Society of Cardiology clinical consensus statement. European Heart Journal.

[104] Sedrak P, Dounaevskaia V, Mancini GBJ, Zieroth S, McKelvie RS, Chiu W (2025). Vaccination in Patients with Cardiovascular Disease: A Case-Based Approach and Contemporary Review. CJC Open.

[105] Wełnicki M, Kamiński K, Mastalerz-Migas A, Kuchar E, Jankowski P, Gil R (2026). Influenza vaccination as part of cardiovascular prevention - expert opinion supported by the Working Group on Cardiovascular Pharmacotherapy and Working Group on Prevention and Epidemiology Section of the Polish Cardiac Society. Kardiologia Polska.

[106] Garcia-Zamora S, Pulido L, Sosa Liprandi MI, Nacinovich F, Balsano FJ, Herrera Paz JJ (2026). Consensus document on the role of adult vaccination in the prevention of cardiovascular events. Joint Statement by the Argentine Federation of Cardiology (FAC), Argentine Society of Cardiology (SAC), and the Argentine Council of Cardiology Residents (CONAREC). MEDICINA (Buenos Aires).

[107] Achterbergh RCA, McGovern I, Haag M (2024). Co-Administration of Influenza and COVID-19 Vaccines: Policy Review and Vaccination Coverage Trends in the European Union, UK, US, and Canada between 2019 and 2023. Vaccines.

